# Targeting STAT3 signaling overcomes gefitinib resistance in non-small cell lung cancer

**DOI:** 10.1038/s41419-021-03844-z

**Published:** 2021-05-31

**Authors:** Zhe Liu, Liang Ma, Yiming Sun, Wenying Yu, Xue Wang

**Affiliations:** 1 Department of pharmacy, the First Affiliated Hospital of Bengbu Medical College, Bengbu, 233004 China; 2 College of pharmacy, Bengbu Medical College, Bengbu, 233030 China; 3 State Key Laboratory of Natural Medicines, China Pharmaceutical University, Nanjing, 211198 China; 4 Wuxi School of Medicine, Jiangnan University, Wuxi, 214122 China

**Keywords:** Lung cancer, Drug development

## Abstract

Lung cancer is one of the most aggressive cancers with poor prognosis and high resistance rate. The family of signal transducer and activator of transcriptions (STATs) appears to modulate resistance in non-small cell lung cancer (NSCLC). In this work, we demonstrated that STAT3/ZEB1 is a critical axis in gefitinib resistance. STAT3-targeted inhibition therefore is a new potential therapeutic strategy for gefitinib resistance in lung cancer. Our small molecule screening identified a relatively specific STAT3-targeted inhibitor, LL1. Pharmacological and biochemical studies indicated that LL1 block the activation of STAT3 via inhibiting its phosphorylation. Further in vitro and in vivo studies elucidated that LL1 sensitizes the resistance cells to gefitinib through depleting STAT3 activity and blocking STAT3/ZEB1 signaling pathways. Little toxicity of LL1 was observed in animal models. All these favorable results indicated that LL1 is a chemotherapeutic adjuvant for gefitinib resistance in NSCLC.

## Introduction

Lung cancer is a malignant tumor originating from the bronchial mucosa or lung glands. Globally, the incidence and mortality of lung cancer are increasing. According to statistics from the American Cancer Society, there were 228,150 new cases and 142,670 deaths of lung cancer in America^1^. Among the different types of cancer, lung cancer has been the well-deserved first killer^2^.

Lung cancer is mainly divided into non-small cell lung cancer (NSCLC) and small cell lung cancer (SCLC)^3^. NSCLC accounts for about 80% of the total lung cancer, and various molecular mechanisms such as gene mutation and abnormal expression have been confirmed to be related to the pathogenesis of NSCLC^4^. With the continuous development and progress of targeted drug therapy, a variety of molecular targeted therapeutic drugs have been put into clinical use or are undergoing clinical trials. At present, the main targets of lung cancer targeted drugs that have been marketed and under development at home and abroad include epidermal growth factor receptor (EGFR) (HER1/ERBB1), HER2, MET, ROS1, VEGF, VEGFR2, and ALK^5–10^. Gefitinib is a representative antineoplastics used for patients with EGFR mutation^11,12^. However, gefitinib resistance will appear after 10–12 months treatment, and the underling mechanisms of gefitinib resistance are still not well understood^13^.

STAT3, a member of the STAT family, mainly activated by phosphorylation at Tyr705 of IL-6 receptor, leukemia inhibitory factor receptor, c-MET or non-receptor tyrosine kinases (EGFR)^14–16^. After activation, STAT3 dimerizes through its SH2 domain, transports to the nucleus, and acts as a transcription factor to induce the expression of target gene. The activation of STAT3 is accompanied by the upregulation of cyclin D1, c-Myc, and Bcl-XL, which promotes cell survival and proliferation^17–19^. Dysregulation of STAT3 activity is related to the pathogenesis of a variety of cancers, including breast, colon, cervical, and prostate cancer^20,21^. Multiple evidences also indicate that STAT3 activity is related to the pathogenesis of lung cancer^22^.

Due to the strong correlation between the activation of STAT3 and tumorigenesis, inhibition of STAT3 by genetic or pharmaceutical modalities has been shown to have antitumor effects in vivo and in vitro^23^. Phosphotyrosine peptides with STAT3 SH2 domain binding activity can inhibit the activation of STAT3^24^. Studies have proved the effectiveness of RNA interference in inhibiting STAT3 and its downstream effectors^25^.

Our previous studies have shown that LL1 is a new STAT3 targeting molecule with significant antitumor activity and selectivity^21^. Mechanistically, LL1 interferes with the binding of SH2 domain, a key domain of STAT3 activation. Binding of LL1 to SH2 domain will prevent activated receptors from recruiting STAT3, thereby affecting the phosphorylation process of STAT3. Therefore, LL1 inhibits tumor growth and invasion by blocking STAT3 signaling pathway. LL1 could be a promising therapeutic drug candidate for cancer by inhibiting the STAT3 activation.

In this study, we found that LL1 could reverse the sensitivity of gefitinib-resistant A549 and PC-9 cells through the suppression of STAT3 activity. Meanwhile, siRNA was used to downregulate the expression of STAT3, and the results showed that it inhibited the activation of downstream signals, providing more direct evidence for STAT3 to participate in cell apoptosis. In addition, LL1, as a new STAT3 inhibitor, increases the sensitivity of gefitinib in vitro and in vivo, suggesting that gefitinib combined with STAT3 inhibitors may be considered as an alternative strategy for the treatment of NSCLC patients with acquired resistance to gefitinib. In summary, our research shows that targeting STAT3 may be an effective treatment for gefitinib-resistant NSCLC.

## Results

### STAT3 mediates resistance of lung cancer tissues and cells to gefitinib

**Fig. 1:**
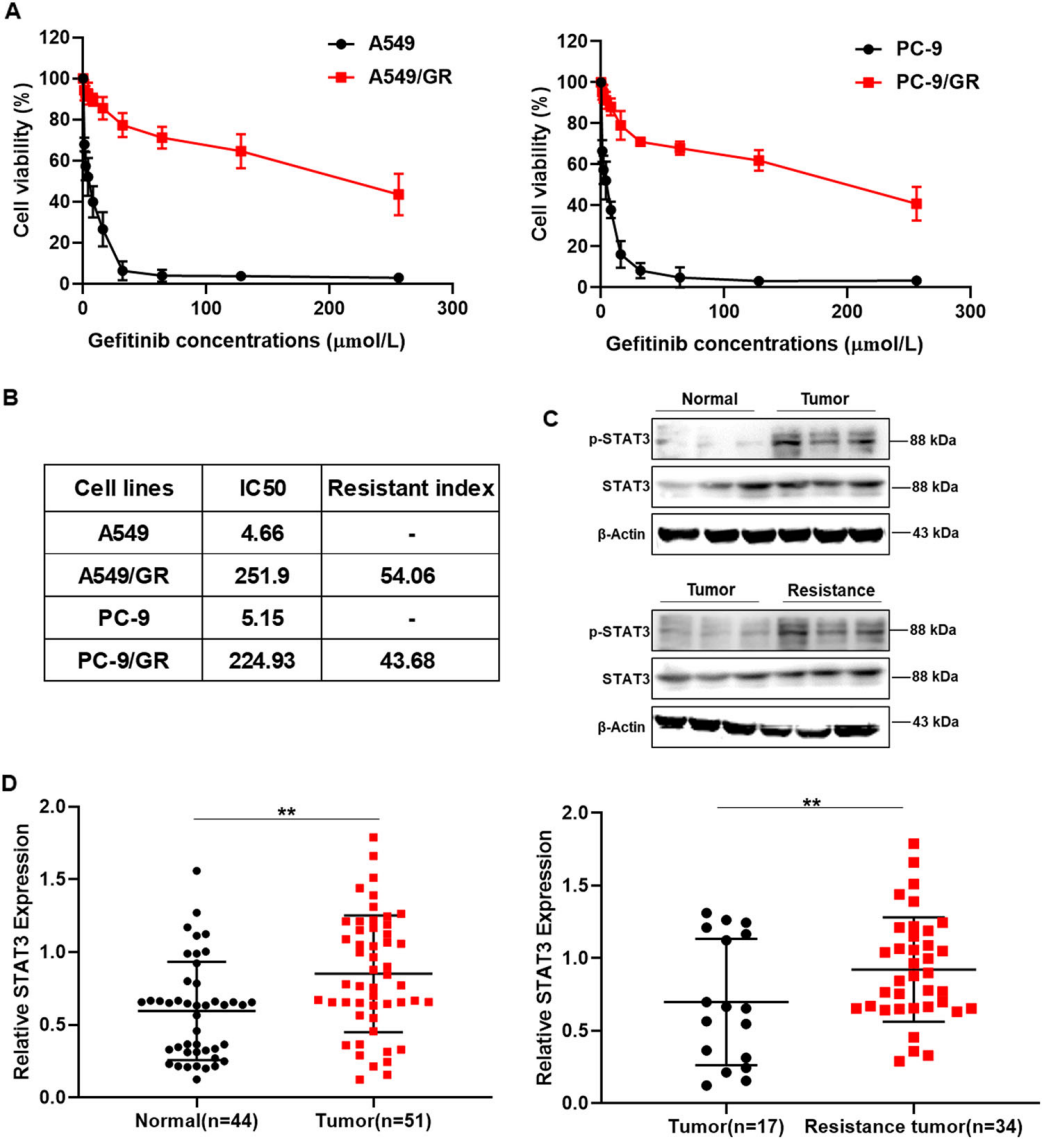
The expression of p-STAT3 was elevated in gefitinib-resistant lung cancer tissues and cells. **A** A549, A549/GR, PC-9, and PC-9/GR cells were treated with the indicated doses of gefitinib for 24 h. Cell viability was determined using the MTT assay. **B** IC50 and resistance index of lung cancer cell lines. **C**, **D** The expression of p-STAT3 in gefitinib-resistant lung cancer patients was upregulated. The expression levels of the indicated proteins were examined by Western blot analysis. Data were represented as mean ± SD, *n* = 3. **p < 0.01, significantly different.

In order to explore the resistance of gefitinib in lung cancer, we built gefitinib resistant A549 and PC-9 cell lines, named A549/GR and PC-9/GR, and used MTT assay to detect the sensitivity of A549/GR and PC-9/GR cells to gefitinib. The results demonstrated that the half-maximal inhibitory concentration (IC_50_) were significantly rised compare to parental A549 and PC-9 cells, and the resistance index (RI) of A549/GR and PC-9/GR cells were 54.06 and 43.68 (Fig. 1A, B). To determine the molecular mechanism of resistance, we analyzed data of gene microarray between resistant cells and parent cells. The results showed that STAT3 expression were significantly elevated, and a variety of STAT3 related genes exhibited obviously differential expression (Supplemental Table 1). Subsequently we detected the expression of p-STAT3 and STAT3 in lung cancer patients-derived tissue. The results demonstrated that the expression of p-STAT3 is significantly increased in lung cancer tumor, especially in resistance tumor tissues (Fig. 1C, D). These data above indicated that STAT3 maybe the critical target of gefitinib resistance.

### STAT3 promotes lung cancer resistance to gefitinib

**Fig. 2:**
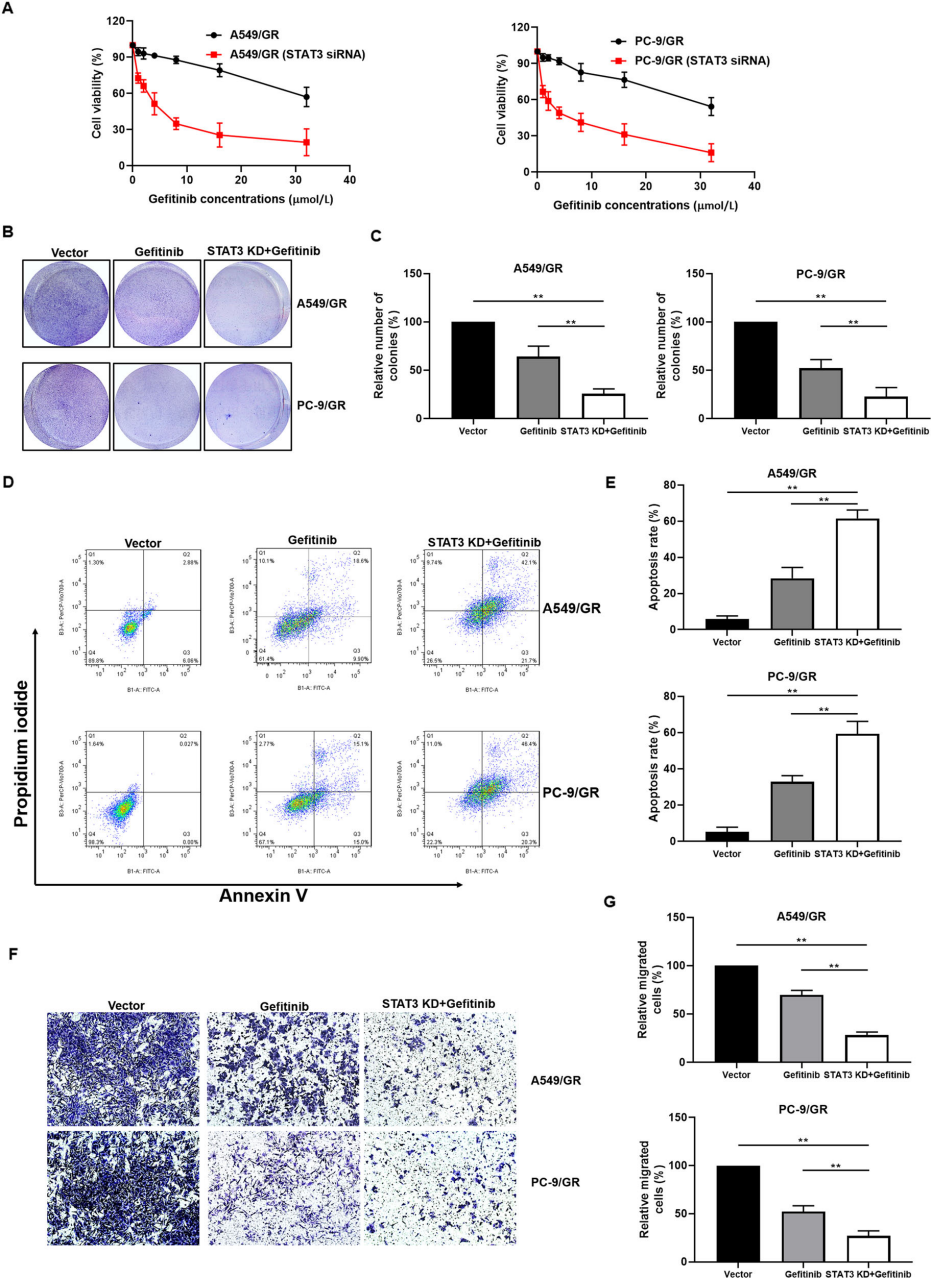
STAT3 knockdown sensitized lung cancer cells to gefitinib. **A** Downregulation of STAT3 increases the sensitivity of lung cancer cells to gefitinib. Cell viability was determined using the MTT assay. **B** A549/GR and PC-9/GR cells were seeded in six-well plates, incubated overnight, and treated with gefitinib (16 μmol/L) or transfected with STAT3 siRNA plus gefitinib (16 μmol/L) for 7 days. Effect of STAT3 siRNA and gefitinib on cell proliferation determined by colony formation assay. **C** Quantification of colony formation. **D** Flow cytometry analysis of apoptotic death of A549/GR and PC-9/GR cells. Cells were treated with either drug alone (16 μmol/L gefitinib) or in combination with STAT3 siRNA for 24 h, and stained with Annexin V/PI. **E** Quantification of PI and Annexin V staining. **F** Effect of gefitinib (16 μmol/L) or gefitinib (16 μmol/L) plus STAT3 siRNA on A549/GR and PC-9/GR cell invasion was determined by transwell assay. **G** Quantification of transwell. The Data were mean ± SD, *n* = 3. **p value <0.01, significantly different.

The previous results found that STAT3 is importantly related to gefitinib resistance, therefore we further explore the molecular mechanism of STAT3 induced gefitinib resistance. We manipulated the expression of STAT3 in A549/GR and PC-9/GR cell lines by siRNA and plasmid (Supplemental Fig. 1A), and we found that STAT3 regulates the cell biological function in gefitinib resisitance lung cancer cells (Supplemental Fig. 1B, D). Furthermore, downregulation of STAT3 enhanced the sensitivity of A549/GR and PC-9/GR cells to gefitinib (Fig. 2A). Compare to the cells treated with gefitinib alone, STAT3 knockdown significantly compromised colony formation capacity in the presence of gefitinib in A549/GR and PC-9/GR cells (Fig. 2B, C). In order to explore the effect of STAT3 on cell biology, we detected the apoptosis of A549/GR and PC-9/GR via PI and Annexin V staining. Knockdown of STAT3 in A549/GR and PC-9/GR significantly increased the proportion of apoptosis cell following the treatment of gefitinib (Fig. 2D, E). In addition, we evaluated the effect of STAT3 knockdown on cell invasion and migration. The transwell results showed that downregulation of STAT3 inhibited the invasion and migration in A549/GR and PC-9/GR cells (Fig. 2F, G). These data indicated that increased STAT3 level promotes the resistance of lung cancer cells.

### ZEB1 involved in STAT3 induced-gefitinib resistance

**Fig. 3:**
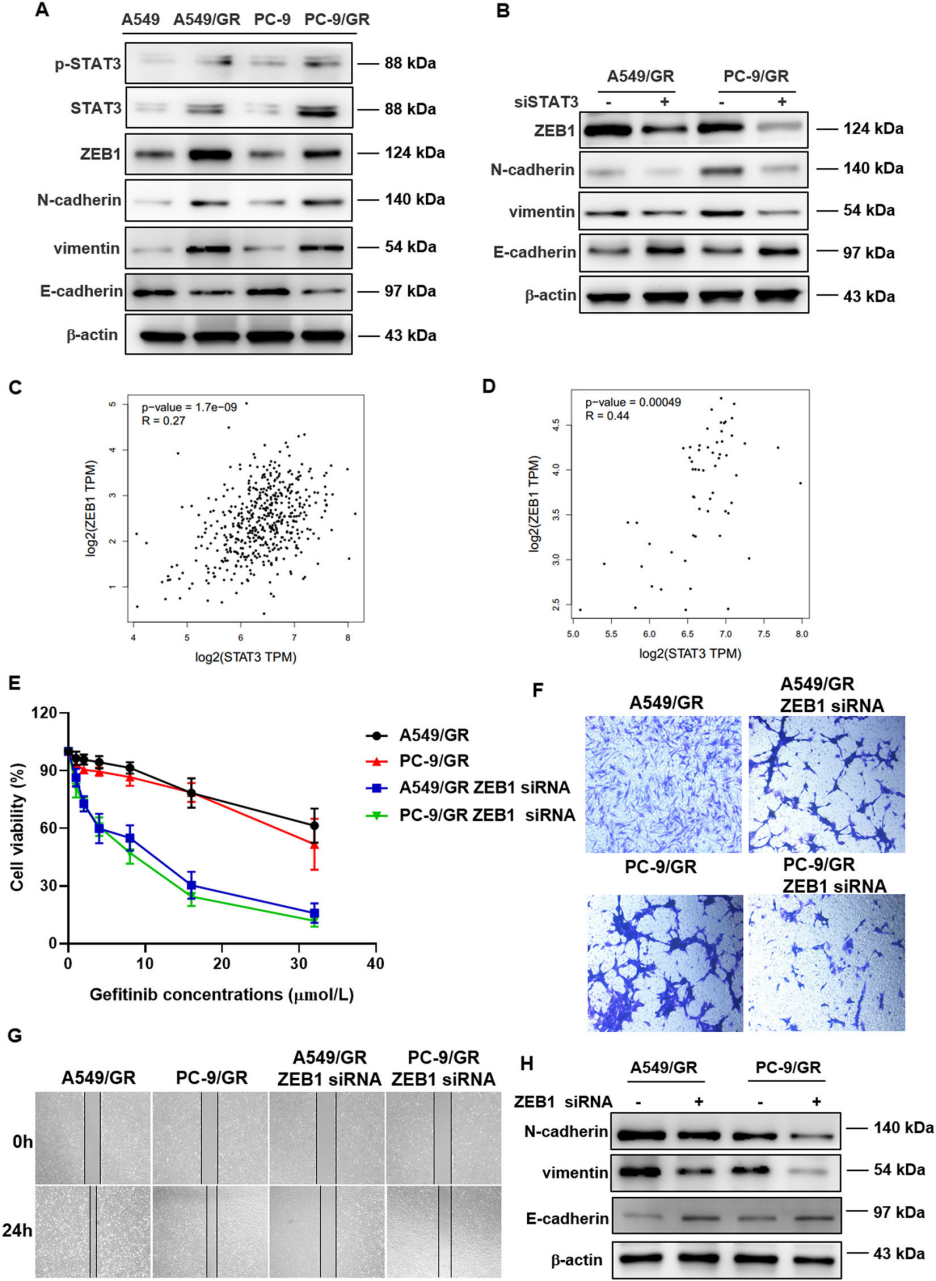
Identification of ZEB1 as the mediator involved in the therapeutic effects conferred by STAT3 inhibition. **A** Protein levels of ZEB1, E-cadherin, vimentin, and N-cadherin were detected by western blot in A549, A549/GR, PC-9, and PC-9/GR cells. **B** STAT3 regulated the expression of ZEB1, E-cadherin, vimentin, and N-cadherin. The expression levels of the indicated proteins were examined by Western blot. **C** Correlation analysis between STAT3 and ZEB1 in tumor tissue. **D** Correlation analysis between STAT3 and ZEB1 in normal tissue. **E** Downregulation of ZEB1 increases the sensitivity of lung cancer cells to gefitinib. Cell viability was determined using the MTT assay. Downregulation of ZEB1 inhibits cell invasion (**F**) and migration (**G**). **H** ZEB1 regulated the expression of E-cadherin, vimentin, and N-cadherin. The expression levels of the indicated proteins were examined by Western blot.

We had identificated STAT3 as a critical target in gefitinib resistance, however the signaling axis is still undefined. As a signal transduction and transcription activator, STAT3 is responsible for a series of downstream gene signals. We tried to find the mediator involved in STAT3 induced-gefitinib resistance. The above results showed that STAT3 regulated the cell invasion and migration, we detected the expression of genes related to invasion and migration. The results demonstrated that the expression of ZEB1, N-cadherin, and vimentin increased, and E-cadherin level declined (Fig. 3A). Silencing STAT3 lead to upregulation of E-cadherin and downregulation of ZEB1, N-cadherin, and vimentin (Fig. 3B). Through analyzing the TCGA database via the Gene Expression Profiling Interactive Analysis (GEPIA), we found that the expression of STAT3 were correlated with ZEB1 in both lung cancer tissues and normal tissues (Fig. 3C, D). Moreover, silencing ZEB1 sensitized A549/GR and PC-9/GR cells to gefitinib (Fig. 3E). The wound healing and transwell data demonstrated that silencing ZEB1 inhibits the invasion and migration of gefitinib-resistant lung cancer cells (Fig. 3F, G). In addition, silencing ZEB1 cancel STAT3-induced E-cadherin, N-cadherin, and vimentin level regulation (Fig. 3H). These results indicated that gefitinib resistance may occur through STAT3/ZEB1 signaling pathway.

### LL1 specified block the activation of STAT3

**Fig. 4:**
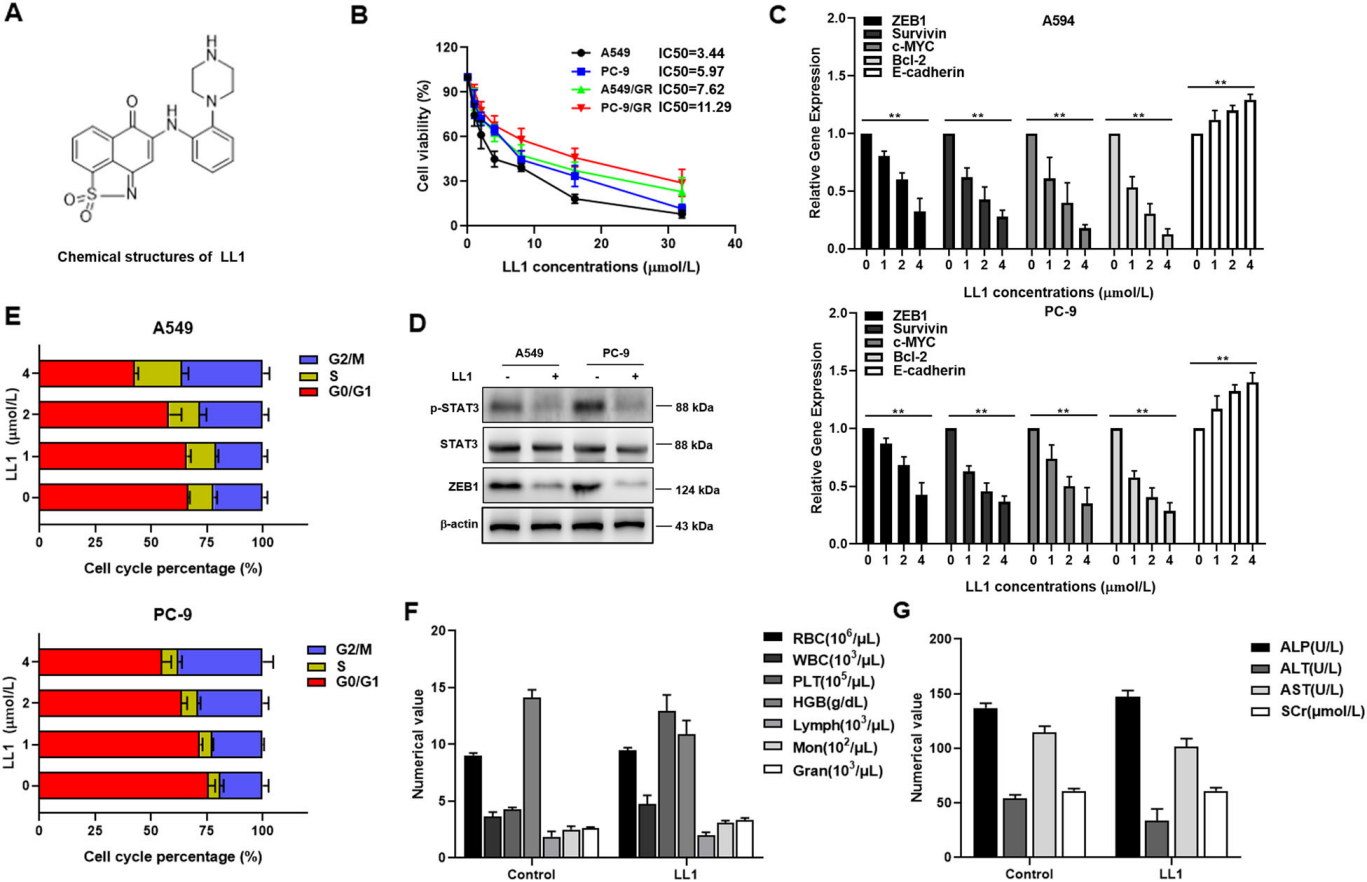
LL1 specified block the activation of STAT3. **A** Chemical structures of novel molecules of LL1. **B** A549 and PC-9 cells were treated with the indicated doses (0, 1, 2, 4, 8, 16, 32 μmol/L) of LL1 for 24 h. Cell viability was determined using the MTT assay. **C** qRT-PCR analysis of ZEB1, Bcl-2, c-myc, E-cadherin and survivin in A549 and PC-9 cells after LL1 treatment for 24 h. Data were mean ± SD, *n* = 3. **p value < 0.01. **D** Protein levels of ZEB1, p-STAT3, and STAT3 were detected by western blot in A549 and PC-9 cells treated with LL1 (2 μmol/L). **E** The percentage of cell cycle distribution for A549 and PC-9 cells treated with indicated concentrations of LL1. **F**, **G** Complete blood count and biochemical tests in the control or LL1 (5 mg/kg) treated mice.

Since STAT3 silence sensitized A549/GR and PC-9/GR cells to gefitinib treatment, we sought to discover an inhibitor targeting STAT3. LL1 (Fig. 4A) is a novel small molecular STAT3 inhibitor, and it specifically binds to STAT3 protein. Following the treatment of LL1, cell viability was markly decreased in a dose-dependent manner (Fig. 4B), the mRNA level of ZEB1, survivin, c-myc, and bcl-2 was downregulated, and E-cadherin was upregulated in A549 and PC-9 cells (Fig. 4C). Moreover, LL1 inhibited the expression of p-STAT3 and ZEB1 (Fig. 4D). Further results showed that LL1 caused G2/M cycle arrest in both A549 and PC-9 cells in a dose-dependent manner (Fig. 4E). It is worth noting that LL1 induces apoptosis and inhibits colony formation in both parental cells and resistant cells (Supplemental Fig. 2A, B). In order to evaluate the safety of LL1 in vivo, we detected its toxicity towards blood, heart, liver, spleen, and kidney in mice. All the blood cell indices were maintained within the normal ranges following LL1 treatment (Fig. 4F). Following the stimulation of LL1, blood biochemical parameters (ALT, AST, ALP, and SCr) showed no significant changes (Fig. 4G). In addition, the viscera weight indices suggested that LL1 had no significant toxicity toward main organs (Data not shown).

### The combination of gefitinib and LL1 reverses resistance in lung cancer cells

**Fig. 5:**
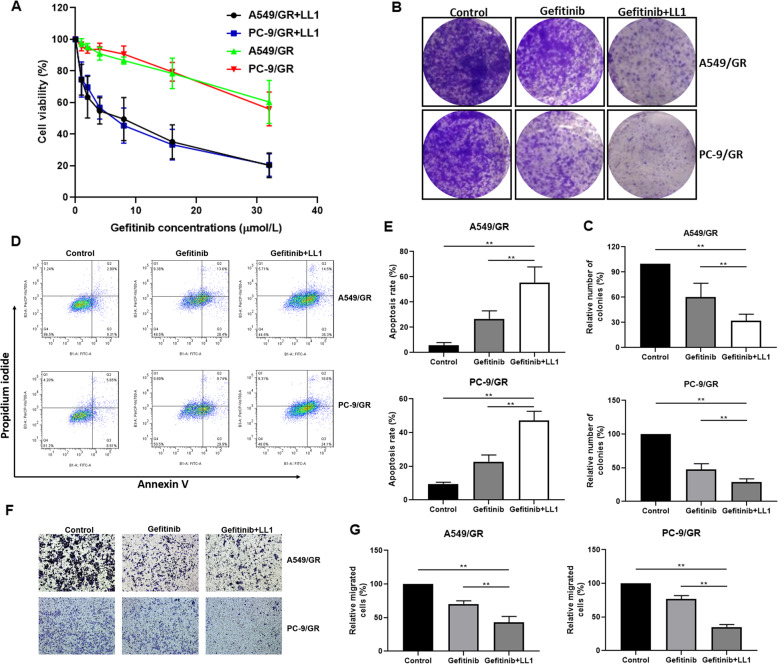
The combination of gefitinib and LL1 reverses resistance in lung cancer cells. **A** LL1 sensitizes the A549/GR and PC-9/GR cells to gefitinb in vitro. Cell viability was determined using the MTT assay. **B** A549/GR and PC-9/GR cells were seeded in six-well plates, incubated overnight, and treated with gefitinib (16 μmol/L) or in combination with LL1 (2 μmol/L) for 7 days. Effect of LL1 and gefitinib on cell proliferation determined by colony formation assay. **C** Quantification of colony formation. **D** Flow cytometry analysis of apoptotic death of A549/GR and PC-9/GR cells. Cells were treated with either drug alone (16 μmol/L gefitinib) or in combination with LL1 (2 μmol/L) for 24 h, and stained with Annexin V/PI. **E** Quantification of PI and Annexin V staining. **F** Effect of gefitinib (16 μmol/L) or gefitinib (16 μmol/L) plus LL1 (2 μmol/L) on A549/GR and PC-9/GR cell invasion was determined by transwell assay. **G** Quantification of transwell. The Data were mean ± SD, *n* = 3. **p value < 0.01, significantly different.

To further confirm whether LL1 sensitizes A549/GR and PC-9/GR cells to gefitinib, we investigated the proliferation, apoptosis, and invasion of lung cancer following the treatment of LL1 in combination with gefitinib. LL1 in combination with gefitinib significantly suppressed the proliferation in A549/GR and PC-9/GR than treated with gefitinib alone (Fig. 5A and Supplemental Table 2). Additionally, the cells exhibited stronger colony formation inhibition upon LL1 + gefitinib treatment (Fig. 5B, C).While treatment with gefitinib alone induced some level of cell death in A549/GR and PC-9/GR cells, LL1 in combination with gefitinib induced more cell death, as shown by Annexin V and PI staining (Fig. 5D, E). Moreover, compare to the treatment with gefitinib or LL1 alone, LL1 plus gefitinib induced more invasion suppression in A549/GR and PC-9/GR cells (Fig. 5F, G).

### The combination therapy of gefitinib plus LL1 potently inhibits tumor progression in vivo

**Fig. 6:**
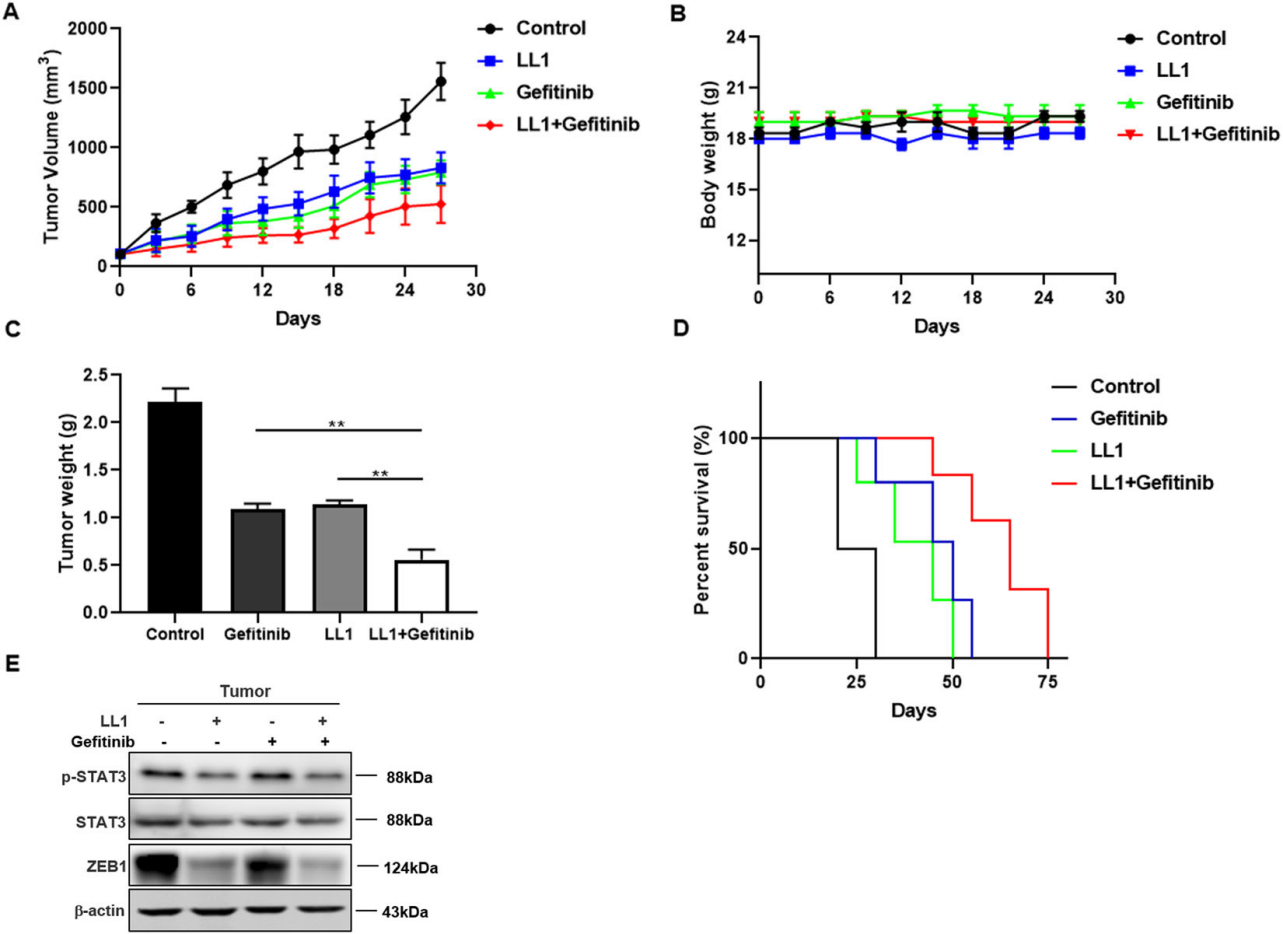
The combination therapy of gefitinib plus LL1 potently inhibits tumor progression in vivo. **A** The effect of LL1, gefitnib, LL1 plus gefitnib or vehicle on tumor volume of subcutaneous A549/GR xenografts in nude mice (*n* = 6). **B**, **C** Body weight and tumor weight from indicated treatment groups. **D** Survival curves (*n* = 6 per group). **E** The expression of ZEB1, STAT3, and p-STAT3 were examined by Western blot. Data were means ± SD relative to control group. **p < 0.01.

Given the in vitro findings, we further evaluated the in vivo antitumor efficacy of LL1 in combination with gefitinib on A549/GR cells in xenograft model. A549/GR cell-derived xenograft tumors in the combined drug group (LL1 + gefitinib) had lower volumes than did those treated with LL1 or gefitinib alone and the overall survival rate of LL1 plus gefitinib group was higher than that of LL1 or gefitinib group (Fig. 6A, B). Furthermore, the weights of nude mice did not fluctuate significantly during the treatment of LL1 (Fig. 6C), but the weights of tumor in combined drug group showed obvious less than in LL1 or gefitinib group (Fig. 6D). To further confirm the effectiveness of LL1 in vivo, we detected the expression of p-STAT3, STAT3, and ZEB1. ZEB1 and p-STAT3 were decreased significantly in tumors (Fig. 6E).

## Discussion

**Fig. 7:**
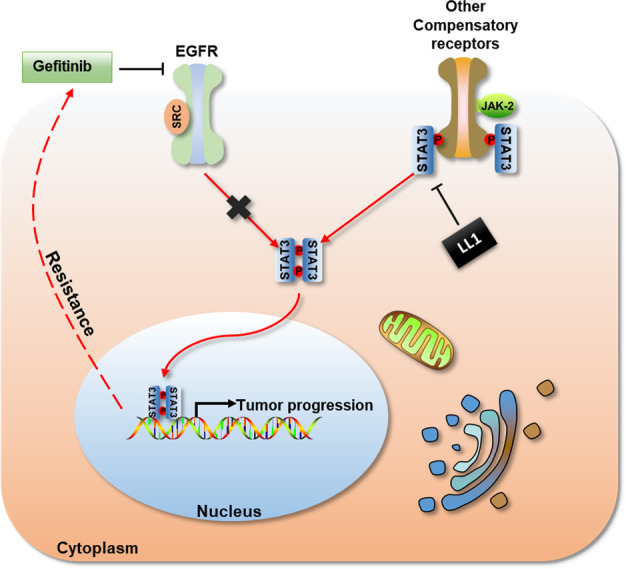
Schematic diagram of STAT3 in gefitinib resistance. When gefitinib resistance occurs, the cancer cells would try to break through the EGFR/STAT3 signaling pathway. Due to the persistent inhibition of EGFR by gefitinib, they had to compensatively activate other signaling pathways which pass through STAT3. Blocking STAT3 activation by LL1 can reactivate the efficacy of gefitinib.

Although the diagnostic and therapeutic approaches had made significant advances, the 5-year survival rate of lung cancer still remains less than 15%, especially in patients with advanced lung cancer. The available options for patients with advanced lung cancer are almost only radiotherapy and chemotherapy which is poorly effective. The emergence of targeting drugs had made progresses in the treatment of lung cancer. However, drug resistance is the most important factor restricting the clinical treatment effect of targeting drugs^26^. Gefitinib is a selective EGFR tyrosine kinase inhibitor which hinder tumor growth, metastasis, and angiogenesis, and increase tumor cell apoptosis^27,28^. Gefitinib is widely used in the treatment of NSCLC, nevertheless high proportion eventually develop acquired resistance. Approximately 50% of the resistant cases have been detected with T790M mutation, but the reason of the rest cases remains uncertain^29,30^. Previous studies suggested that lung cancer cells can produce gefitinib resistance by compensatoryly increasing the activity of other signaling pathways. When the patients were treated with gefitinib for a long time, the EGFR/STAT3 signaling pathway was persistently inhibited. At the same time, the body would try to clear the EGFR/STAT3 signaling pathway. However, due to the inhibition of EGFR by gefitinib, the body had to compensatively activate other signaling pathways which pass through STAT3 (such as Src/STAT3 and Jak2/STAT3), resulting in the increased STAT3 expression. In this study, we identified STAT3 axis as the critical target to regulate gefitinib resistance. Moreover, we identified STAT3 inhibitor, LL1 as a synthetic chemical agent against lung cancer and comfirmed LL1 plus gefitinib combinational treatment strategy for gefitinib resistance (Fig. 7).

Signal transducer and activator of Transcription 3 (STAT3) has been recognized as an oncogene that promotes cell proliferation, apoptosis, metastasis, and immune escape^31–33^. Growing evidences suggested that aberrant activation of STAT3 induces malignant progression of cancer. Persistent STAT3 activity has been discovered in many cancers, such as breast cancer, colorectal cancer, liver cancer, lung cancer, etc^34–36^. Moreover, the expression level of p-STAT3 is linked to the prognosis of cancer, suggesting that STAT3 could be a promising target for cancer^37^. In fact, there were already evidences that STAT3 level has a clear relationship with resistance in cancer^38^. Recent study reported that increased STAT3 level affects cetuximab resistance in patients with colorectal cancer^39^. Similarly, another study demonstrated that the patients who failed to response to gefitinib were detected low levels of p-EGFR but high levels of p-STAT3^40^. These studies suggested that inhibition of STAT3 may be required for targeted therapy in cancer.

Generally, the association of cytokines to their receptors (such as IL-6R, LIFR, c-MET, EGFR, and so on) induces the dimerization of receptor molecules, subsequently JAKs coupled to the receptors approach each other and activated by interactive tyrosine phosphorylation. The activated JAKs catalyzes tyrosine phosphorylation of the receptor to form the STAT3 docking site. STAT3 binds to the receptor through SH2 domain and phosphorylated by JAKs. The phosphorylated STAT3 form homo/heterodimers and translocate into nucleus. Finally bind to the promoter of target gene, activating transcription. It should be noticed that STAT3 is the main downstream target of both EGFR and c-MET^41,42^. Previous studies suggested that c-MET is significantly activated while gefitinib resistance occurs, indicating STAT3 play a critical role in gefitinib acquired resistance^43^.

In the present study, we constructed gefitinib-resistant A549 and PC-9 lung cancer cell lines. Microarray analysis demonstrated that STAT3 signaling activity elevated in A549/GR and PC-9/GR cells. The A549/GR and PC-9/GR cells exhibited increased ability of migration, and this phenomenon may relate to the upregulation of p-STAT3, ZEB1, N-cadherin, and Vimentin. In addition, we further confirmed in clinical samples that the expression of p-STAT3 is elevated in gefitinib-resistant lung cancer tissues. In order to validate the contribution of STAT3 in gefitinib-resistance, we silenced the expression of STAT3 in A549/GR and PC-9/GR cells. We found that knockdown of STAT3 obviously enhanced the sensitivity of A549/GR and PC-9/GR cells to gefitinib. Moreover, silence STAT3 increased the apoptosis and colony formation inhibition induced by gefitinib in A549/GR and PC-9/GR cells, and inhibited the invasion and migration in A549 and PC-9 cells. It may be inferred that the activation of STAT3 mediates the resistance of gefitinib in lung cancer.

Although persist active STAT3 may induce cancer progression and gefitinib resistance, the mechanisms remained unclear. We have comfirmed that silencing STAT3 may affact in cell proliferation, apoptosis, invasion, and migration, therefore we focus on the downstream target. Recently, ZEB1 has been reported to promote human breast tumorigenesis and metastasis through ZEB1/p53 signaling axis^44^. In present study, we found silencing STAT3 could decrease the expression of ZEB1 and ZEB1-related genes (N-cadherin and vimentin). Furthermore, silencing ZEB1 canceled the gefitinib-resistance reverse induced by STAT3 knockdown, indicating that gefitinib-resistance may regulated via STAT3/ZEB1 axis.

In view of the ability of reduced STAT3 to reverse drug resistance, adjuvants targeting STAT3 could be used to optimize the chemotherapeutic effects of gefitinib. LL1 is a novel STAT3 small molecule inhibitor discovered and synthesized through computer-aided design. LL1 inhibits STAT3 activation by binding to SH2 domain of STAT3 protein. Recent studies have shown that LL1 suppresses the growth of colorectal cancer by block the activation of STAT3. In our study, LL1 was used to inhibit STAT3 activity. We found that LL1 in combination with gefitinib indeed sensitized A549/GR and PC-9/GR cells following the treatment of gefitinib, and exhibited great effectiveness in the inhibition of A549/GR cell-derived tumor formation in animal models. Furthermore, no obvious toxicity observed in mice blood, indicating the safety of LL1 in vivo. Therefore, the combination of LL1 and gefitinib provides a new strategy for relieving clinical gefitinib resistance in NSCLC.

In summary, the present study revealed that gefitinib resistance in NSCLC may be promoted via STAT3/ZEB1 signaling pathway. Abnormal activation of STAT3 promotes cells proliferation and invasion in lung cancer. When gefitinib blocks EGFR, lung cancer may further stimulate tumor growth through compensatory receptors. However, multiple signaling pathways converge in STAT3, leading to a critical role in tumor growth and resistance. Therefore, STAT3 inhibition could be a promising strategy to provide more effective clinical treatment of gefitinib resistant NSCLC. LL1, a novel STAT3 inhibitor significantly sensitize lung cancer cells to gefitinib in vitro and in vivo. These findings provide the preclinical evidence required for the resistance in NSCLC, and further clinical studies are essential to validate LL1 as a chemotherapeutic adjuvant for gefitinib resistance in NSCLC.

## Materials and methods

### Cell lines and chemicals

LL1 was synthesized in Wenying Yu’s laboratory, and the purity is greater than 98%. Both LL1 and Gefitinib used in the experiment were dissolved in DMSO, and the final percentages of DMSO were less than 0.5%. Human A549 and PC9 cells line were purchased from American Type Culture Collection (ATCC, Manassas, Virginia). Cells maintained in DMEM medium supplemented with 10% fetal bovine serum (Gibco, American), 100 μg/mL penicillin, and 100 μg/mL streptomycin (Thermo Fisher Scientific, USA). All the cell lines were cultured in a 5% CO_2_-humidified atmosphere at 37 °C. Gefitinib used in functional validation experiments were purchased from Selleck Chemicals (USA).

### MTT assay

Cells were plated in 96-well plates overnight and incubated with the designated compounds. After compounds treatment, the MTT (Beyotime Biotechnology, China) was used to detect the cell viability at the time point of 24 h. Add 10 μl of MTT to each well and incubate at 37 °C for 4 h. The absorbance was measured at 490 nm using PowerWavex (BioTEK instruments, USA). The percentage of cell viability was calculated as the absorbance ratio of the treated cells to the DMSO-treated cells.

### Establish drug-resistant cell lines

Culture cells with the medium containing gefitinib at a start concentration of one-tenth of the IC50. Increase the concentration of gefitinib every 2 to 3 weeks, and each increase of about 1.2–1.5 times the dose. Maintain the medication pressure for 6–10 months. Calculate the RI (above 20) via MTT assay.

### Colony formation assay

The cells were seeded in six-well plates at 3000 cells per well. After cultured for 24 h, the cell culture medium was changed to fresh medium with low serum and drugs. After incubating for 10 days, the medium was removed and washed the cells twice with PBS. The cells were fixed with cold paraformaldehyde for 20 min. After stained with crystal violet for 10 min, the cells were washed, dried, and counted.

### Flow cytometric analysis

Cells were plated in 12-well plates overnight and incubated with the designated compounds. After staining for 15 min, the samples were subjected to flow cytometry FACS Calibur detection (BD Bioscience, USA), and Cell Quest Pro software (BD Bioscience, USA) were used to analyze the apoptotic population. Cells in the early stage of apoptosis were positive for Annexin V-FITC, negative for PI, and both staining were positive for the cells in the late stage of apoptosis.

### Western blot

Total cell lysates were prepared using RIPA buffer supplemented with protease inhibitors (Halt Protease inhibitor cocktail, Thermo Scientific) and 40 μg/ml PMSF (Sigma). Cell extracts were resolved in 10% SDS–PAGE and transferred to PVDF membrane (PerkinElmer, Waltham, MA). The membranes were blocked by 5% skim milk in TBS with 0.1% of Tween-20 (TBST) for 2 h at room temperature prior to be incubated overnight with primary antibodies. The primary antibodies including E-cadherin (ab40772), N-cadherin (ab280375), Vimentin (ab92547), and ZEB1 (ab276129) were purchased from Abcam (USA). Phospho-STAT3 (Tyr705) (#9145) and STAT3 (#4904) were obtained from Cell Signaling Technology (USA). The membranes were than washed thrice in TBST and incubated with horseradish peroxidase-conjugated secondary antibodies for 1 h. After consecutive washes, the membranes were visualized using a chemiluminescence kit (PerkinElmer, USA).

### The in vivo gefitinib-resistant NSCLC xenograft model

This study was performed according to guidelines approved by the Bengbu Medical College Institutional Animal Care and Use Committee. Previously, an in vivo gefitinib-resistant NSCLC model (*n* = 6) was generated in-house. Four- to five-week-old female athymic nude mice were purchased from Vital River Laboratory. The mice were randomly stratified into four groups: (1) vehicle control; (2) 50 mg/kg gefitinib; (3) 5 mg/kg LL1; and (4) 50 mg/kg gefitinib and 5 mg/kg LL1. Once the tumor volumes reached ~25 mm^3^, mice were treated by oral gavage with vehicle control (dimethyl sulfoxide 5%, normal saline 50%, and PEG400 50%), gefitinib and/or LL1. Body weights and tumor measurements were performed twice a week and tumor volume was calculated based on the formula: length × width^2^ × 0.5. At the end of the experiment, mice were sacrificed prior to removal of the tumors for further analysis.

In addition, we also evaluated the toxicity of LL1 in vivo. In brief, the mice were treated with LL1 (5 mg/kg, once daily), or vehicle control for 14 days (*n* = 6). Blood samples were then collected from the retro-orbital plexus, and the main organs were excised, weighed, and fixed in 4% paraformaldehyde for further analysis. Complete blood counts were performed by sysmex XE2000 (Sysmex, Kobe, Japan), while plasma biochemical indices were analyzed by Beckman Coulter AU5800 (Beckman Coulter, California, USA).

### RNA interference

Gefitinib-resistant cells were transfected with plasmid or siRNA using Lipofectamin RNAiMAX (Invitrogen, USA) following the manufacturer’s instructions. STAT3 and negative universal control siRNA were purchased from Gema (China). The plasmid of STAT3 was purchased from Genomeditech (China). Expression levels of STAT3 were analyzed by Western blotting as the indicated time STAT3 siRNA transfection. Human STAT3 siRNA and negative control siRNA (Real gene) were transfected into the cells using lipofectamine2000 (Invitrogen, USA) according to the manufacturer’s instruction. Cells were incubated for 48 h before harvested and lysed for protein analysis or processed to cell viability assay.

### STAT3 siRNA sequences

STAT3#1 [sense (5′-3′): GGGACCUGGUGUGUGAAUUAUTT; antisense (5′-3′): AUAAUUCACACCAGGUCCCTT].

STAT3#2 [sense (5′-3′): CCCGGAAAUUUAACAUUCUTT; antisense (5′-3′): AGAAUGUUAAAUUUCCGGGTT].

STAT3#3 [sense (5′-3’′): GGUACAUCAUGGGCUUUAUTT; antisense (5′-3′): AUAAAGCCCAUGAUGUACCTT].

### Transwell assay

Thaw the matrigel (BD Bioscience, USA) overnight at 4 °C. Dilute the matrigel 1:8 with chilled serum-free growth medium just prior to coating. To generate a homogenous mixture pipette up and down slowly, ensuring that no bubbles are introduced into the mixture. The chilled diluted matrigel (70 μl) is placed onto the center of the upper chamber. Place plates in the incubator for 60 min to allow gelling. The cells (1 × 10^5^ per chamber) were seeded in serum-free medium into the upper chamber, and add medium supplemented with 15% FBS to the lower chamber. After incubation for 48 h, migrated cells pass through the membrane and attach to the lower chamber side of the membrane. The migrated cells were stained with crystal violet and counted.

### Wound healing assay

The cells (5 × 10^5^ per well) were seeded in six-well plates. When the cells reached 100% confluence, the cells were scratched using a yellow tip and washed twice to remove non-adherent cells. The cells were allowed to migrate into the scratched area for 24 h, and observed under the microscope.

### Cell cycle analysis

The cells (1 × 10^5^ per well) were seeded in 12-well plates. After culture overnight, the cells were treated with various doses of LL1. After 24 h, the cells were collected and fixed with 75% alcohol. DNA was labeled with PI/RNAse staining buffer (Beyotime Biotechnology, China) for 20 min in the dark at 4 °C. The cell cycle was measured by MACSQuant X after PI dying with PI/RNase staining buffer (BD Bioscience, USA).

### Quantitative RT-PCR

Total mRNA was isolated from cells using TRIzol (Invitrogen, USA) and cDNA was synthesized using HiScript Q RT SuperMix for qPCR (Vazyme Biotech, China). Quantitative RT-PCR (qRT-PCR) analysis was performed using SYBR green on an iCycler mounted with an iQ5 Multicolor Real-Time PCR Detection System (Bio-Rad, USA). The relative gene expression level between treatments was calculated using the following equation: relative gene expression = 2^−(ΔCtsample−ΔCtcontrol)^.

### Sequences

ZEB1 (F): 5′-GCCAATAAGCAAACGATTCTG-3′

ZEB1 (R): 5′-TTTGGCTGGATCACTTTCAAG-3′

Bcl-2 (F): 5′-GGTGGGGTCATGTGTGTGG-3′

Bcl-2 (R): 5′-CGGTTCAGGTACTCAGTCATCC-3′

C-MYC (F): 5′-CTCGAATTCCTTCCAGATATCCTCGCTG-3′

C-MYC (R): 5′-CACTGCGCGCTGCGCCAGGTTT-3′

Survivin (F): 5′-AGGACCACCGCATCTCTACAT-3′

Survivin (R): 5′-AAGTCTGGCTCGTTCTCAGTG-3′

E-cadherin (F): 5′-GACGCGGACGATGATGTGAAC-3′

E-cadherin (R): 5′-TTGTACGTGGTGGGATTGAAG-3′

β-Actin (F): 5′-AGCGAGCATCCCCCAAAGTT-3′

β-Actin (R): 5′-GGGCACGAAGGCTCATCATT-3′.

### Statistical analysis

All data are expressed as mean values ± standard error of the mean (SEM). Mean values in xenograft studies were compared by one way analysis of variance (ANOVA) to determine the significance, *p < 0.05, **p < 0.01.

## Supplementary information

Supplemental information

Supplemental Figure 1

Supplemental Figure 2

Supplemental Table 1

Supplemental Table 2

uncropped Fig.1D

uncropped Fig.3A

uncropped Fig.3B

uncropped Fig.3H

uncropped Fig.4D

uncropped Fig.6E
