# Supplementary material for: Targeting STAT3 signaling overcomes gefitinib resistance in non-small cell lung cancer
Source: Cell Death Dis. 2021 May 31;12(6):561. doi: 10.1038/s41419-021-03844-z (PMC8166856; doi:10.1038/s41419-021-03844-z)
Supplement: Supplementary file 1 — Supplemental information [file 41419_2021_3844_MOESM1_ESM.docx]

**SUPPLEMENTAL INFORMATIONS**

**Targeting STAT3 signaling overcomes gefitinib resistance in non-small cell lung cancer**

Zhe Liu^1, 2^, Liang Ma^1, 2^, Yiming Sun^1, 2^, Wenying Yu^3^, Xue Wang^4^

^1^ Department of pharmacy, the First Affiliated Hospital of Bengbu Medical College, Bengbu 233004, China

^2^ College of pharmacy, Bengbu Medical College, Bengbu 233030, China

^3^ State Key Laboratory of Natural Medicines, China Pharmaceutical University, Nanjing 211198, China

^4^ Wuxi School of Medicine, Jiangnan University, Wuxi, China.

Running title: Targeting STAT3 in lung cancer

Conflict of Interest statement: There is no conflict of interest

Correspondence should be address to:

Yiming Sun, Department of pharmacy, the First Affiliated Hospital of Bengbu Medical College, Bengbu, 233004, China; Email: 924295535@qq.com; Wenying Yu, State Key Laboratory of Natural Medicines, China Pharmaceutical University, Nanjing, 211198, China; Email: ywy@cpu.edu.cn; Xue Wang, Wuxi School of Medicine, Jiangnan University, Wuxi 214000, China; Email: 909121969@qq.com.

**EXPERIMENTAL SUPPLEMENTAL PROCEDURES**

**Microarray processing and analysis**

The RNA of A549 and A549/GR cells were extracted using Trizol reagents. The GeneChip® Human Genome U133 Plus 2.0 array (Affymetrix, Santa Clara, CA, USA) was used for microarray processing to determine gene expression profiling depending on the manufacturer’s instructions. Samples were then scanned in an Affymetrix 3000 scanner. Significant differentially expressed genes between A549 cells and A549/GR cells were selected based on the following criteria: P value <0.05 and absolute fold change >1.3.

**Molecule screening of LL1**

Computer-aided drug design is widely utilized for the efficient identification and optimization of lead compounds. With the aim of designing druggable STAT3 inhibitors, the multiple ligand simultaneous docking (MLSD) method was advanced in two aspects, i.e., fragment library design and linker design. Using advanced MLSD (AMLSD), we designed 26 compounds that directly inhibit both phosphorylation and dimerization of STAT3 protein. In order to investigate the selectivities of these compounds for STAT1/3/5 in silico, the compounds were docked to the crystal structures of STAT1, STAT3 and STAT5. Limited by the docking data and the availability of the starting materials, we chose 5 componds for further biological tests. According to Lipinski’s rules and the results of biological tests, LL1 exhibited the best activity and druggability.

**Clinical samples**

Lung cancer tissue (including adenocarcinoma and squamous carcinoma) and corresponding adjacent normal tissues were gathered from non-small cell lung cancer patients at the First Affiliated Hospital of Bengbu Medical College. All tissues were collected during surgery, and stored at -80℃ after being washed by phosphate buffered saline. Cut the sample of 100-200mg, grind it with liquid nitrogen in a mortar. Sieve and lysate the sample. Store in aliquots at -80℃ for subsequent testing.

**Supplemental figure legends**

**Supplemental Figure 1: STAT3 regulates the cell biological function in gefitinib resisitance lung cancer cells.**

A. PC-9/GR and A549/GR cells were transfected with STAT3 siRNA or cDNA. After 48 h, the expression of STAT3 and p-STAT3 was determined by western blot.

B. Effect of STAT3 siRNA and cDNA on cell proliferation determined by colony formation assay.

C. Effect of STAT3 siRNA and cDNA on cell apoptosis determined by PI & Annexin V staining.

D. Effect of STAT3 siRNA and cDNA on cell invasion determined by transwell.

**Supplemental Figure 2: LL1 inhibits the colony-formation and induces apoptosis in lung cancer cell lines**

A. A549, PC-9, PC-9/GR and A549/GR cells were treated with LL1 (2 μM) for colony-formation assay.

B. A549, PC-9, PC-9/GR and A549/GR cells were treated with LL1 (2 μM) for 24 h and prepared for PI & Annexin V staining assay.

**Supplemental Table 1: Affymetrix Array analysed data of A549 & A549/GR cells.**

**Supplemental Table 2: Combination index (CI) of LL1 and gefitinib combination in A549/GR and PC-9/GR cells.**
