# Supplementary figures and images for: Targeting STAT3 signaling overcomes gefitinib resistance in non-small cell lung cancer
Source: Cell Death Dis. 2021 May 31;12(6):561. doi: 10.1038/s41419-021-03844-z (PMC8166856; doi:10.1038/s41419-021-03844-z)

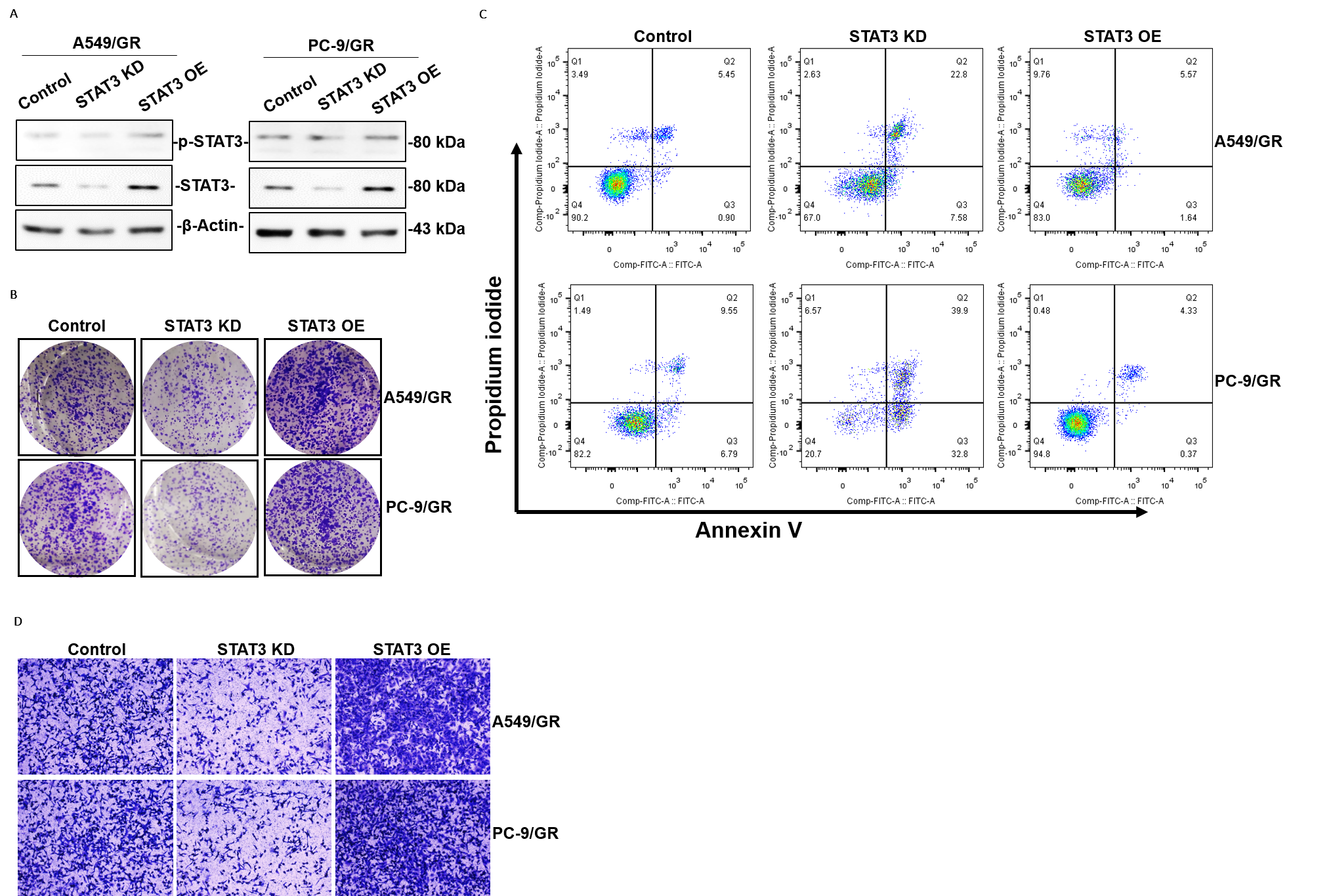

Supplement: Supplementary file 2 — Supplemental Figure 1 [file 41419_2021_3844_MOESM2_ESM.png]

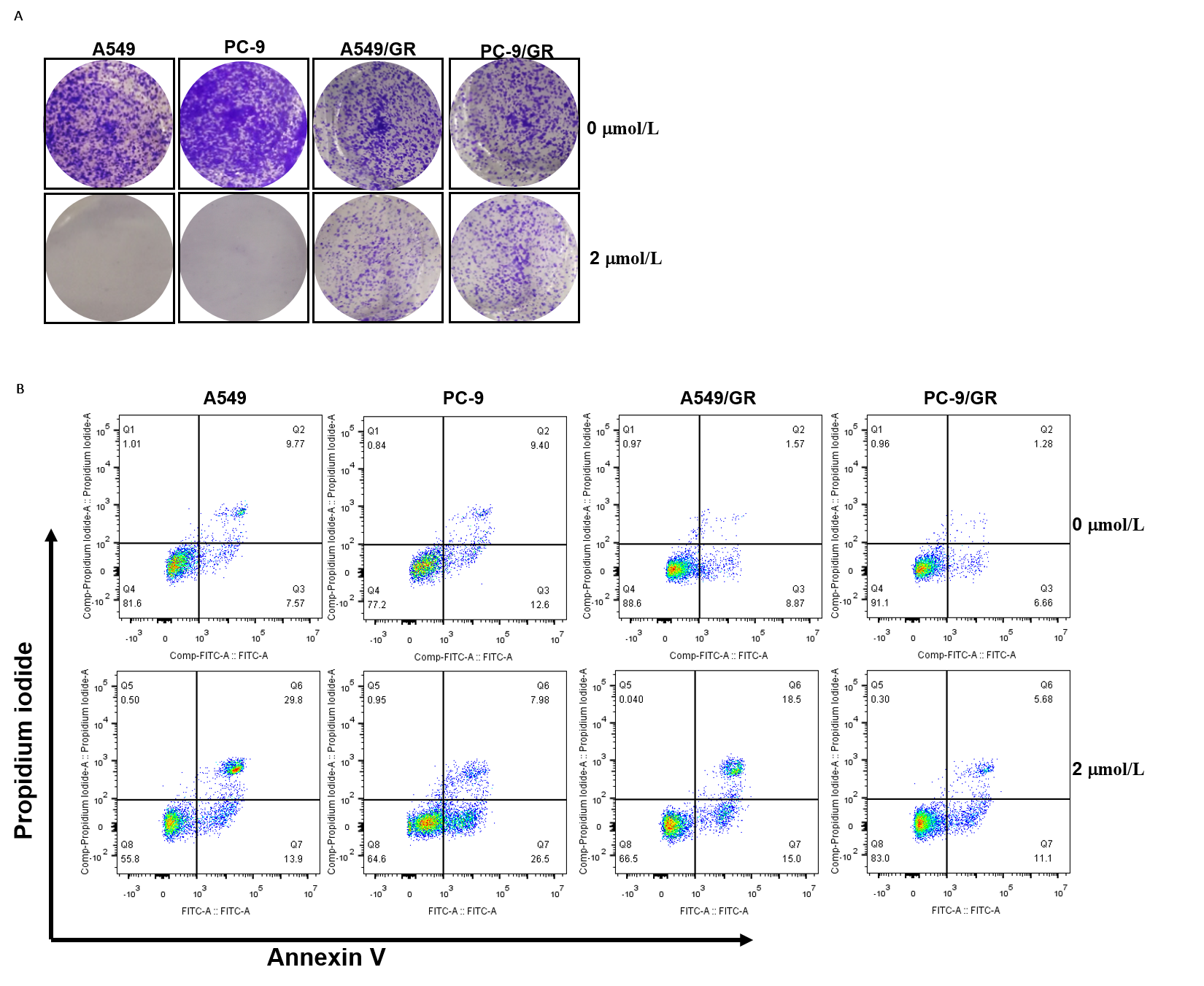

Supplement: Supplementary file 3 — Supplemental Figure 2 [file 41419_2021_3844_MOESM3_ESM.png]

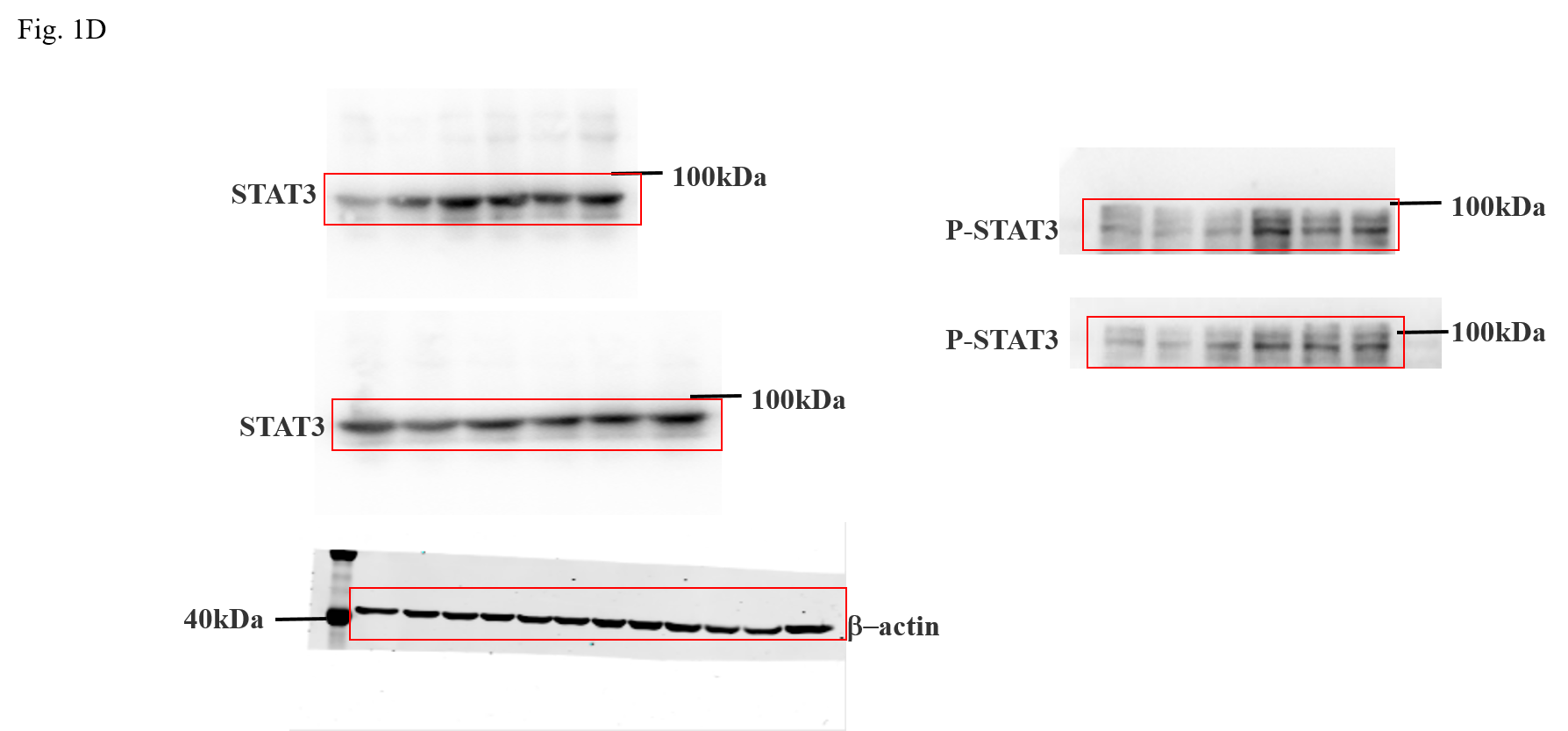

Supplement: Supplementary file 6 — uncropped Fig.1D [file 41419_2021_3844_MOESM6_ESM.png]

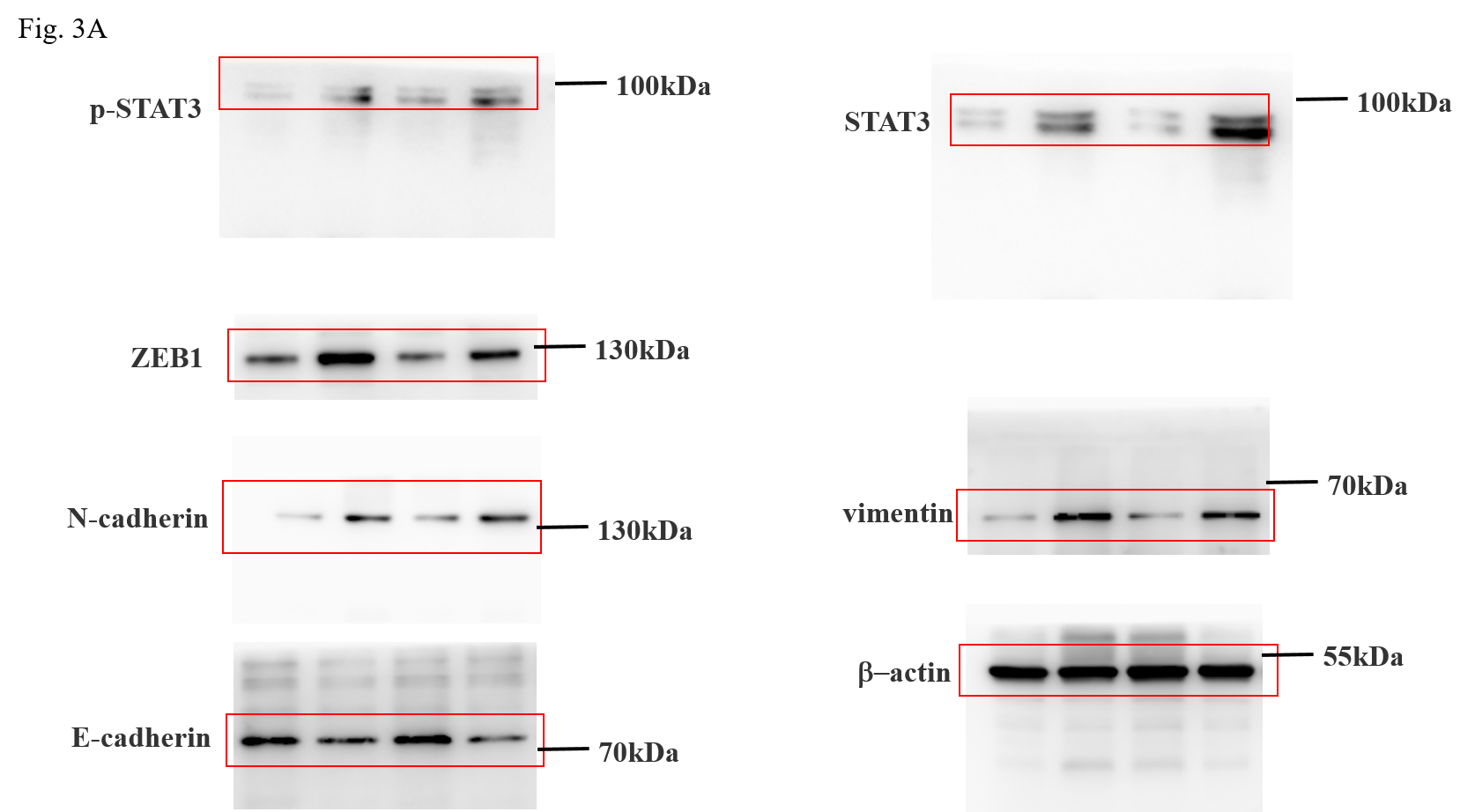

Supplement: Supplementary file 7 — uncropped Fig.3A [file 41419_2021_3844_MOESM7_ESM.png]

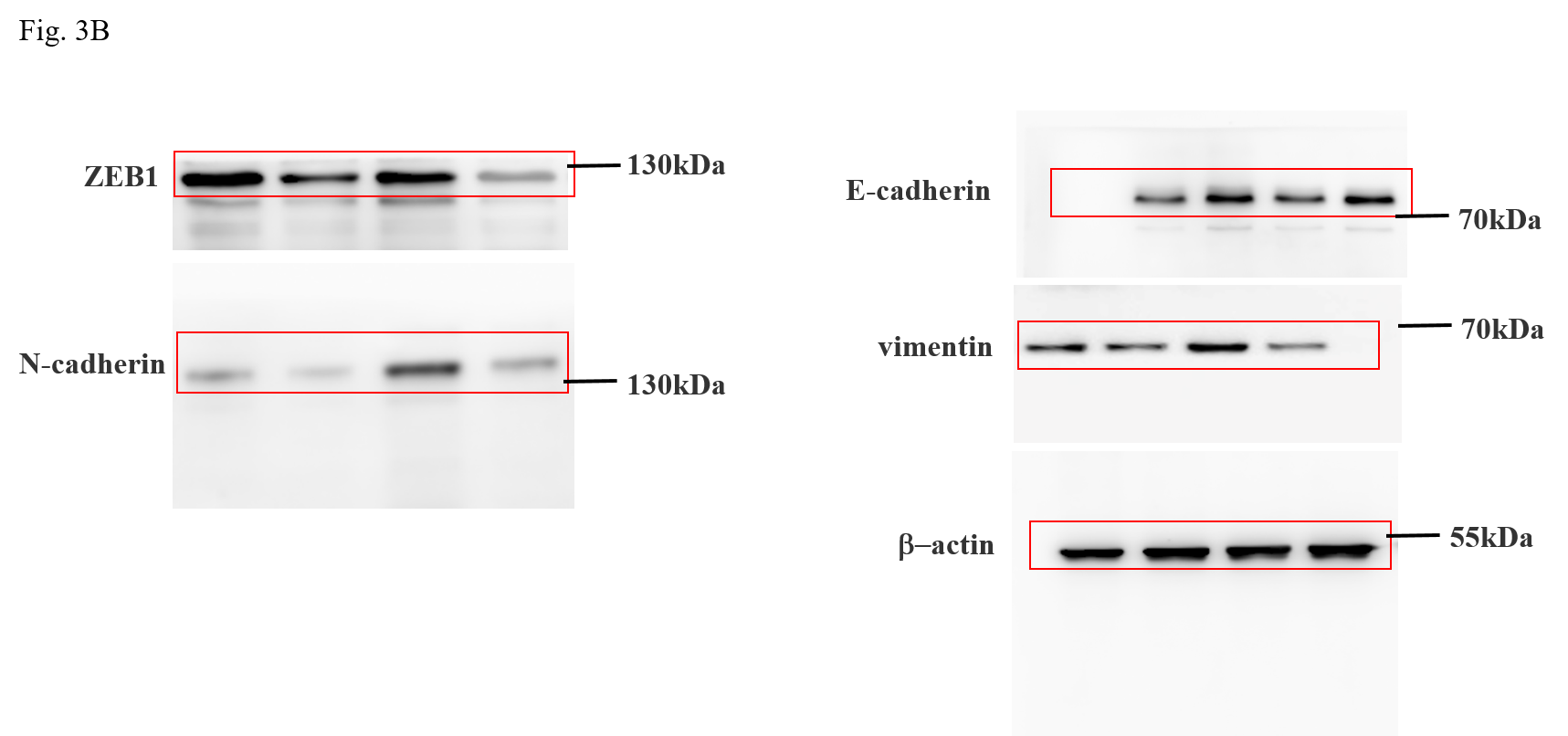

Supplement: Supplementary file 8 — uncropped Fig.3B [file 41419_2021_3844_MOESM8_ESM.png]

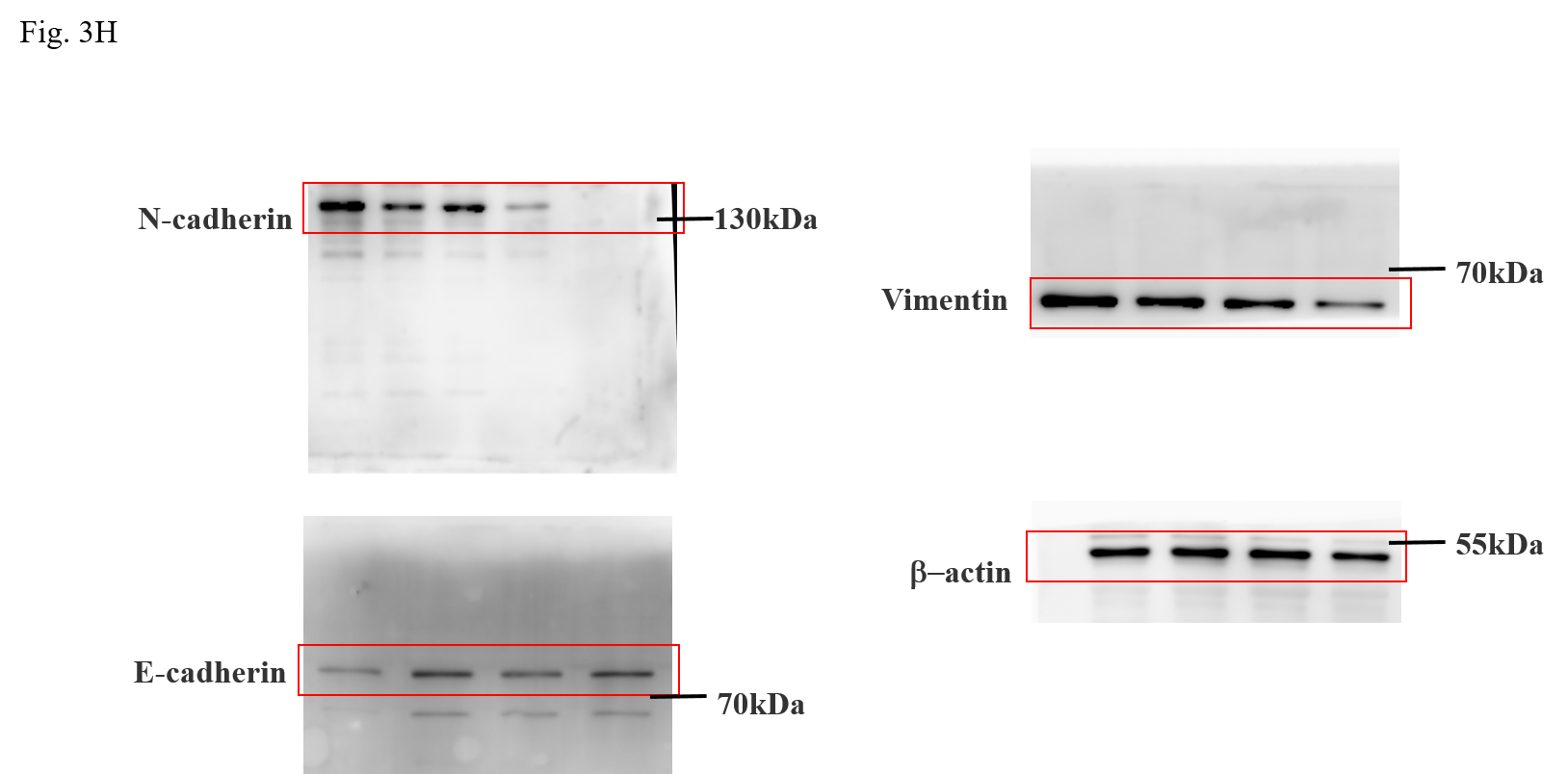

Supplement: Supplementary file 9 — uncropped Fig.3H [file 41419_2021_3844_MOESM9_ESM.png]

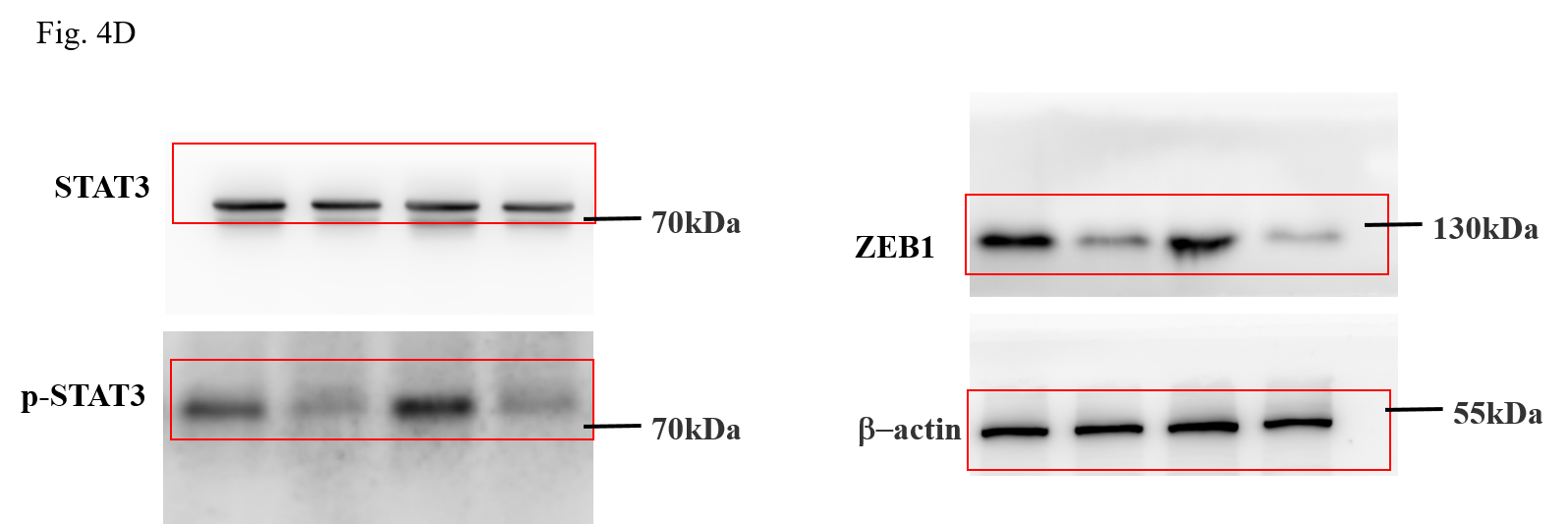

Supplement: Supplementary file 10 — uncropped Fig.4D [file 41419_2021_3844_MOESM10_ESM.png]

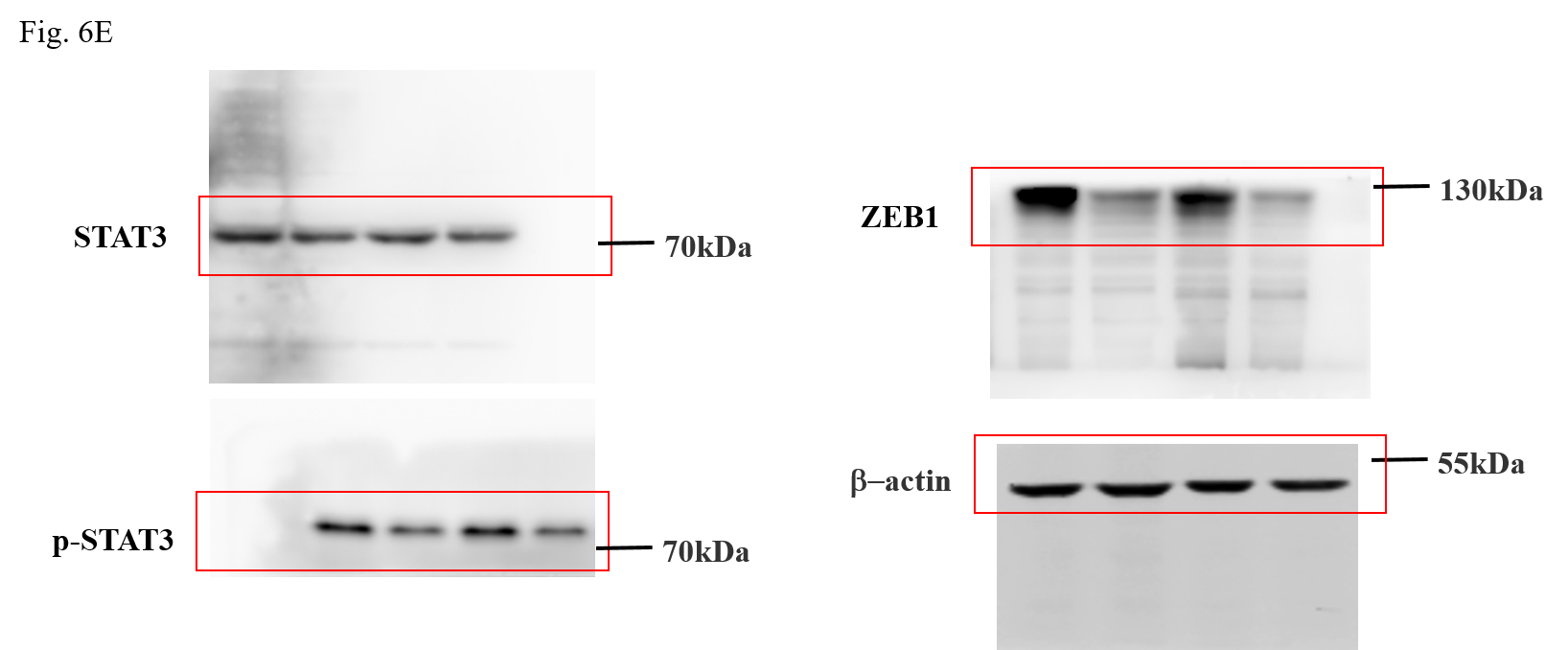

Supplement: Supplementary file 11 — uncropped Fig.6E [file 41419_2021_3844_MOESM11_ESM.png]
